# Let-7f-5p Inhibits PRRSV Replication by Regulating Lipid Metabolic Reprogramming in Infected Cells

**DOI:** 10.3390/vetsci12121176

**Published:** 2025-12-10

**Authors:** Dongfeng Jiang, Jin Huang, Xiaotong Wang, Guangwei Zhao, Congcong Li, Liyu Yang, Xiangge Meng, Qiuliang Xu

**Affiliations:** 1 College of Animal Science and Technology, Henan University of Animal Husbandry and Economy, Zhengzhou 450046, China; jdf13526758315@163.com (D.J.); 251001@hnuahe.edu.cn (J.H.); wangxiaotong@webmail.hzau.edu.cn (X.W.); guangweizhao001@163.com (G.Z.); 80813@hnuahe.edu.cn (C.L.); 231037@hnuahe.edu.cn (L.Y.); 2 Henan Research Institute of Pig Breeding and Industry Development, Zhengzhou 450046, China

**Keywords:** pig, PRRSV, lipid metabolism, SREBP2, let-7f-5p

## Abstract

Lipids provide essential membrane components and energy sources for viral replication, playing multiple roles in viral infection. However, the mutual influence between lipid metabolism and PRRSV proliferation remains unclear. Using transcriptomics, lipidomics, BODIPY staining, and Western blot (WB) analysis, our findings revealed that PRRSV infection significantly altered the abundance of lipid-metabolism-associated genes and lipid metabolites in cells. qRT-PCR confirmed that PRRSV infection dose-dependently upregulated SREBP2 expression (*p* < 0.01), while BODIPY staining demonstrated a significant increase in intracellular lipid droplets post-infection (*p* < 0.01). Let-7f-5p significantly reduced lipid droplet accumulation and suppressed PRRSV N protein expression. Notably, 15 lipid species that were upregulated during PRRSV infection were downregulated by let-7f-5p overexpression. These lipids were enriched in pathways related to phosphatidylcholines, monounsaturated fatty acids, and C16-C18 fatty acid metabolism. Exogenous palmitic acid (C16:0) treatment reversed the inhibitory effects of let-7f-5p on *SREBP2* expression and viral replication, demonstrating that viral proliferation can be regulated by modulating host lipid metabolism. This study reveals that PRRSV hijacks host lipid metabolism to facilitate viral replication, whereas let-7f-5p exerts antiviral effects through dual mechanisms. These findings provide new insights into host-directed antiviral strategies against PRRSV infection.

## Simple Summary

Altered cellular metabolism is a hallmark of viral infection, and changes in lipids also impact viral replication. However, the mutual influence between lipid metabolism changes and PRRSV proliferation remains unclear. The objective of this study was to investigate the alterations in lipid metabolism in PK15^CD163^ cells before and after viral infection, as well as the effects of cellular lipid metabolism changes on PRRSV proliferation. We found that PRRSV infection induces host lipid metabolic reprogramming, while inhibiting SREBP2 expression and reducing lipid droplet formation can suppress PRRSV proliferation.

## 1. Introduction

Porcine reproductive and respiratory syndrome (PRRS) is a highly immunosuppressive disease characterized by miscarriage, stillbirths in sows, as well as severe respiratory diseases in piglets [1,2], The porcine reproductive and respiratory syndrome virus (PRRSV), which causes the disease, demonstrates significant genetic diversity and a high mutation rate; the PRRSV strain that emerged in 2006 especially demonstrates enhanced virulence traits, including rapid transmission, high mortality, increased genetic variability, potent immunosuppression, persistent infection, and prolonged viral shedding, posing a greater threat to the global swine industry [3]. Therefore, the research on the mechanism of PRRSV infection and prevention methods is still an urgent task.

PRRSV belongs to the arteritis virus family of single-stranded, positive-stranded RNA viruses, and its viral particles are spherical in shape, with diameters of about 50–70 nm, and have an enveloped structure [4]. The PRRSV genome is approximately 15 kilobases long and contains 11 open reading frames, including a 5′ untranslated region (UTR) and a 3′ poly(A) tail [5,6]. The 5′ region encodes 16 nonstructural proteins (nsps) involved in immune evasion through ubiquitination modulation, immune pathway suppression, and autophagy manipulation [7,8], while the 3′ region produces eight structural proteins mediating viral entry via Cluster of Differentiation 163 (CD163), heparan sulfate (HS), porcine sialoadhesin (pSn) and vimentin (VIM) receptors [9,10].

Lipids maintain cellular homeostasis through membrane formation, signaling and energy production [11]. The SREBP (Sterol Regulatory Element-Binding Protein) family is the cellular lipid homeostatic “core switch” and consists of three isoforms, SREBP-1a, SREBP-1c, and SREBP-2, with SREBP-1a and SREBP-1c encoded by the SREBP-1 gene and SREBP-2 encoded by a separate gene [12]. It has long been recognized that SREBP1a is involved in overall lipid synthesis, SREBP1c in fatty acid synthesis, and SREBP2 in cholesterol regulation [13,14]. However, by constructing SREBP2 gene-deficient mice, Vergnes found that SREBP2 deficiency not only affects the expression of cholesterol biosynthesis genes, but also significantly reduces the expression of SREBP1a, SREBP1c, and inhibits the expression of fatty acid synthesis genes [15]. Viruses commonly reprogram host metabolism to facilitate replication [16]. For example, the Newcastle disease virus promotes glucose utilization and aerobic glycolytic pathways in infected cells [17], human cytomegalovirus enhances glucose and glutamine uptake [18], and Kaposi’s sarcoma-associated herpesvirus infection enhances amino acid metabolism [18]. Lipids play a role in viral entry into host cells, both directly and indirectly. They are involved in the fusion process between the viral envelope and host cell membranes by regulating membrane fluidity, autophagy, β-oxidation and ATP production. Consequently, the reprogramming of lipid metabolism is a common feature of viral infections [19]. PRRSV infection also requires lipid involvement. Long et al. found that fatty acids are required for PRRSV replication, but the organism in turn inhibits fatty acid synthesis and suppresses viral replication by activating the AMP-activated protein kinase (AMPK)-acetyl-CoA carboxylase 1 (ACC1) signaling pathway after viral infection, which may be a defense response of the organism [20]. PRRSV induces the degradation of lipid droplets into free fatty acids by activating the autophagy pathway, which provides energy support for viral replication [21]. In addition, PRRSV can activate lipid synthesis through the reactive-oxygen-species-dependent AKT/PCK1/INSIG/SREBPs signaling axis [22]. Green tea extract epigallocatechin gallate was also found to limit PRRSV replication and assembly by disrupting lipid metabolism [23]. A recent study reported that the Yin Yang 1 transcription factor could regulate intracellular lipid droplet synthesis by altering the expression of the fatty acid synthase gene FASN and the PPARγ receptor, thereby affecting PRRSV replication [24].

As a regulator of viral infection, miRNAs can not only bind to specific viral RNA or DNA sequences, leading to viral gene degradation or translational inhibition, but also indirectly regulate viral replication by shaping the host immune response [25,26]. For example, miR-150 not only directly targets the PRRSV genome to regulate viral replication but also inhibits host suppressor of cytokine signaling-1 expression, suppresses the JAK-STAT pathway, and enhances IFN-I, thereby inhibiting PRRSV replication [27]. let-7 family sequences and functions are highly conserved across species. let-7f-5p, as an important member of its family, plays a key role in various biological processes such as angiogenesis [28], immune cell differentiation [29], and microbial infection [30]. The let-7 family can inhibit PRRSV-2 replication through NEAT1 and ARID3A/NF-κB [31]. Li et al. also found that let-7f-5p could inhibit PRRSV replication by targeting non-muscle myosin heavy chain 9 (MYH9) [25]. Our group previously confirmed that let-7f-5p has a targeting relationship with SREBP2 through dual luciferase experiments, and it can significantly inhibit the expression of SREBP2 and other lipogenic genes [32]. This study aims to utilize transcriptomics and lipidomics technologies to comprehensively analyze the impact of PRRSV infection on cellular lipid metabolism. Furthermore, it employs miRNA transfection targeting key lipid metabolism genes to intervene in the host’s lipid metabolism network and assess its effect on viral replication. This research provides a crucial scientific foundation for developing novel anti-PRRSV therapies based on host metabolic regulation.

## 2. Materials and Methods

### 2.1. Cells and Viruses

PK15 cells and their CD163-expressing derivatives (PK15^CD163^, generously provided by Prof. En-ming Zhou, Northwest A&F University for PRRSV susceptibility studies) were cultured in Dulbecco’s Modified Eagle Medium (DMEM) (Gibco, South Logan, UT, USA), supplemented with 10% fetal bovine serum (FBS) (Gibco, South Logan, UT, USA) at 5% CO_2_ and 37 °C. The PRRSV strain WUH3, supplied by Prof. Shaobo Xiao from Huazhong Agricultural University, Wuhan, China, was used to infect the cells for 1 h at 37 °C. Thereafter, the culture medium was replaced with maintenance medium containing 2% FBS until cell collection.

### 2.2. miRNAs and Transfections

The let-7f-5p (UGAGGUAGUAGAUUGUAUAGUU) mimics and a negative control (NC, UUCUCCGAACGUGUCACGUTT) were synthesized by GenePharma (Shanghai, China). The 50 nM mimics and NC were transfected into cells using Lipofectamine™ 2000 transfection reagent (Invitrogen™, Carlsbad, CA, USA) for subsequent experiments. In brief, cells were seeded in 6-well or 12-well plates, and the medium was replaced with Opti-MEM when the cells with 60% confluent. miRNAs and Lipofectamine 2000 were diluted in Opti-MEM, diluted Lipofectamine 2000 and siRNAs were mixed and incubated for 20 min at room temperature. The siRNA–lipid complex was added to the cells.

### 2.3. Quantitative Real-Time PCR

Lyse the cells with RNAiso Plus (TAKARA, Tokyo, Japan) completely. Then, add chloroform, shake vigorously, and let stand at room temperature. After centrifugation, aspirate the upper aqueous phase into a new tube, add an equal volume of isopropanol, and mix well to form a white RNA precipitate. Wash the RNA precipitate with 75% ethanol, and collect the precipitate by centrifugation. Reverse-transcribe 1 μg of the total RNA into cDNA using a RevertAid First Strand cDNA Synthesis kit (Thermo Fisher Scientific, Carlsbad, CA, USA). The cDNA was subjected to quantitative real-time PCR analysis using TB Green^®^ Premix Ex Taq™ (TAKARA, Tokyo, Japan) on a BioRad CFX384 system (Bio-Rad, Richmond, VA, USA). The thermal cycle program of the quantitative real-time PCR was 30 s at 95 °C, followed by 40 cycles of 95 °C for 5 s, 60 °C for 30 s, followed by the melt curve. After the reaction, the sample cycle quantification (Cq) value is analyzed relative to the standardized GAPDH using the 2^−ΔΔCt^ method. The details of the primers are listed in Table 1.

### 2.4. Western Blot

The total protein of cells was extracted using RIPA lysis buffer (Beyotime, Shanghai, China), PMSF (Beyotime, Shanghai, China). Add 5x sample buffer (Fermentas, Beijing, China) to the protein sample. Boil for 10–15 min to denature the proteins. The protein was subjected to electrophoresis using SDS-PAGE (90 V for 0.5 h and 120 V for an additional hour) and then transferred to polyvinylidene difluoride (PVDF) membrane (Millipore, Burlington, MA, USA). PVDF membranes were blocked with 5% non-fat dry milk for 2.5 h at room temperature and subsequently incubated with primary antibodies overnight at 4 ° C, respectively (Table 2). After being washed with TBST (0.01% Tween-20 in TBS) for 3 times, the membranes were then incubated with corresponding secondary antibodies for 2 h at room temperature. Immunoblot was visualized using ImageQuant LAS4000 mini (GE Healthcare Life Sciences, Piscataway, NJ, USA).

### 2.5. Fluorescent Lipid Droplet Staining

Cells were fixed with 4% paraformaldehyde for 15 min at room temperature. The fixative was aspirated and cells were washed three times with PBS on a shaker at 40 rpm for 5 min each. BODIPY (Thermo Fisher, Waltham, MA, USA) was diluted at a ratio of 1:8000, added to the cells, and incubated for 30 min in the dark. It was washed three times with PBS, and an anti-fade mounting medium was added to the slide. The coverslip was gently removed from the culture plate and mounted with the medium, and air bubbles were carefully eliminated. Stored slides were protected from light at 4 °C until imaging.

### 2.6. Enzyme-Linked Immunosorbent Assay (ELISA) for TNF-α and IL-10

The trypsinized PK-15 or PK-15^CD163^ cells were washed 3 times with PBS, prior to the repeated freezing and thawing in PBS (1 × 10^6^ cells/200 μL) for lysis. After the centrifugation at 3000 rpm for 10 min, the supernatant was gathered, and the TNF-α, IFN-β, and IL-10 levels were quantified using the ELISA kits according to their instructions. In brief, the target-containing sample was added to the microplate coated with the capture antibody and incubated for binding. The plate was washed, and then the enzyme-conjugated antibody was added. It was washed again, the substrate solution was added, the enzyme catalyzed the substrate to produce a color reaction, and then the absorbance was measured.

### 2.7. Transcriptomics Sequencing and Analysis

PK15^CD163^ cells were seeded into 6-well plates, and the cells were infected with PRRSV at a multiplicity of infection (MOI) of 1 for 1 h. The infection medium was then replaced with maintenance medium containing 2% FBS. The cells were then incubated for 36 h at 37 ◦C with 5% CO2, after which the PRRSV-infected PK15^CD163^ cells were harvested for RNA-seq analysis. Total RNA was extracted using RNAI-Plus (Takara, Tokyo, Japan). RNA-seq library construction and sequencing were performed using DNBSEO-T7 with the PE150 model at Bioyi Biotechnology Co., Ltd (Wuhan, China). Subsequently, the raw data were filtered, and the clean data were aligned with the reference genome of *Sus scrofa 11.1* (https://www.ncbi.nlm.nih.gov/assembly/GCF_000003025.6) (accessed on 14 May 2025). The differentially expressed genes (DEGs) between groups were screened by Fold Changes > 2 and FDR < 0.05 based on DESeq2. ClusterProfiler and Metascape (https://metascape.org/gp/index.html, accessed on 11 November 2025) were applied to conduct functional and pathway enrichment (*p*-value < 0.05) for the DEGs.

### 2.8. Lipidomics Analysis

Cell samples were analyzed in this study using lipidomics methods. Cells (1 × 10^7^ cells) in the logarithmic growth phase (70–90% fusion) were first collected, washed with PBS, trypsin-digested and collected, and stored in liquid nitrogen snap-frozen at −80 °C. The samples were separated by HPLC on a Thermo Accucore™ C18 column (2.1 × 100 mm, 2.6 μm), and the mass spectrometry was detected and then quality-controlled by the peak shape of the internal standard, retention time, and signal intensity. Sample reproducibility was assessed using principal component analysis (PCA). Differential lipid screening was combined with univariate analysis (*t*-test/Wilcox test, *p* < 0.05) and multivariate analysis (OPLS-DA, VIP > 1), and the screening criteria were set to be *p* < 0.05, VIP > 1, and|log2FC|> 0. Finally, lipid classification was performed based on the LIPID MAPS database, and functional enrichment was carried out by utilizing Fisher’s exact test and the LION database (http://lipidontology.com/, accessed on 11 May 2025) for functional enrichment analysis to reveal the biological functions of differential lipids.

### 2.9. Statistical Analysis

All data in this study had at least three biological replicates and are expressed as mean ± SD. Comparisons between multiple groups were performed using the Kruskal–Wallis test, and comparisons between two groups were performed using the Wilcoxon test, and significance was calculated. *p*-values < 0.05 and <0.01 were considered significant and highly significant, respectively.

## 3. Results

### 3.1. PRRSV Infection Leads to Host Lipid Metabolism Disruption

#### 3.1.1. PRRSV Infection Affects the Expression of Lipid Metabolism Genes in PK15^CD163^ Cells

**Figure 1:**
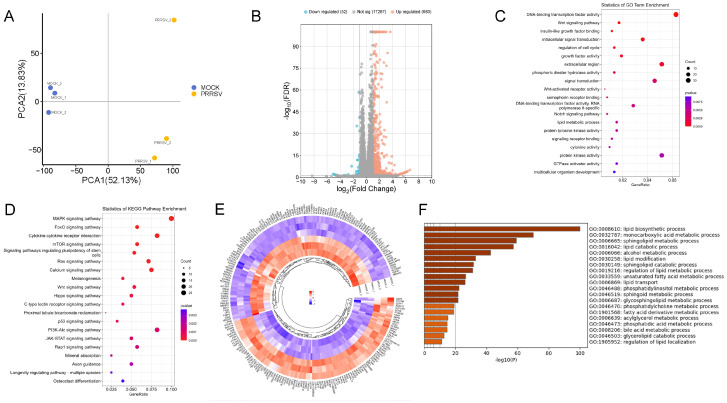
Analysis of differentially expressed genes. (**A**) Principal component analysis plot. (**B**) Volcano plot. (**C**) Differentially expressed gene GO enrichment result. (**D**) Differentially expressed gene KEGG enrichment result. (**E**) Differentially expressed lipid metabolism gene clustering (FDR ≤ 0.05). (**F**) The GO term of differentially expressed lipid metabolism gene clustering (FDR ≤ 0.05).

Sequencing data were analyzed by principal component analysis (PCA), and a more obvious distinction was found between the control and PRRSV-infected group (Figure 1A). Screening of differentially expressed genes with FDR ≤ 0.05 and|log2 (fold change)| ≥ 1 showed that a total of 735 significantly differentially expressed genes were identified after PRRSV infection, of which 683 genes were differentially upregulated and 52 genes were differentially downregulated after PRRSV infection (Figure 1B). GO and KEGG enrichment analyses showed (Figure 1C,D) that the differentially expressed genes were significantly enriched in the Wnt signaling pathway, regulation of cell cycle, signal transduction, lipid metabolic process, MAPK signaling pathway, cytokine–cytokine receptor interaction, calcium signaling pathway, PI3K-Akt signaling pathway, and JAK-STAT signaling pathway. This finding suggests that PRRSV functions similarly to other viruses by activating cellular metabolic pathways to promote cell growth.

Based on 595 lipid-metabolism-related pathway genes included in the Reactome database, the lipid metabolism genes in the experimental data were identified and analyzed. The results showed that a total of 222 lipid metabolism genes differed before and after infection (FDR ≤ 0.05) (Figure 1E). Further analysis of these lipid metabolism genes showed that they were mainly enriched in lipid biosynthetic process, monocarboxylic acid metabolic process, sphingolipid metabolic process, lipid catabolic process, unsaturated fatty acid metabolic process, and other pathways (Figure 1F).

#### 3.1.2. PRRSV Infection Leads to Significant Alterations in Lipid Metabolites in PK15^CD163^ Cells

**Figure 2:**
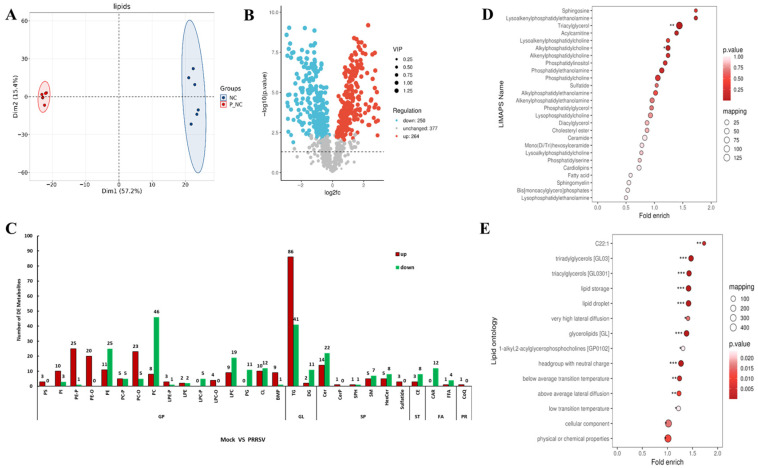
Analysis of cellular differential lipid metabolites before and after PRRSV infection. (**A**) Principal component analysis. (**B**) Differential lipid metabolite heatmap. (**C**) Differential lipids. (**D**) LIPID MAPS functional classification enrichment analysis bubble plots. (**E**) LION lipid enrichment bubble plots. (* *p* < 0.05, ** *p* < 0.01, *** *p* < 0.001).

Lipid sequencing results showed good reproducibility of the samples, which was in line with statistical consistency (Figure 2A). Screening for differential lipid metabolites was performed according to the conditions of *p* < 0.05, and VIP > 1. A total of 514 differential lipid metabolites were identified, of which 264 lipid metabolites were upregulated and 250 lipid metabolism downregulators (Figure 2B). Among them, the expression abundance of lipid small molecules such as triglycerides (TG), ethanolamine plasmalogen (PE-P), ether-linked phosphatidylethanolamine (PE-O), and ether-linked phosphatidylcholine (PC-O) were significantly upregulated, while phosphatidylcholine (PC), ceramide (Cer), lysophosphatidylcholine (LPC), and acylcarnitine (CAR) were significantly downregulated (Figure 2C). The LIPID MAPS enrichment results showed that the differential lipid metabolites were significantly enriched in the metabolic pathways such as TG, PC-O, and CAR in PK15^CD163^ cells infected with PRRSV (Figure 2D). In addition, the LION functional-type enrichment results showed that differential lipid metabolites were not only enriched in key lipid molecules, such as TG and PC-O, but also enriched in cellular component pathways, such as lipid droplets and lipid storage, as well as physical or chemical properties of membrane structure, such as melting point, solubility, charge properties, high lateral diffusivity, and low phase transition temperatures of lipid molecules (Figure 2E). The above results suggest that PRRSV leads to alterations in energy metabolism and membrane fluidity of host cells, causing disorders in host cell lipid metabolism. This result is consistent with the previous results of metabolism genes.

#### 3.1.3. PRRSV Infection Promotes SREBP2 mRNA Expression and Lipid Droplet Increase

**Figure 3:**
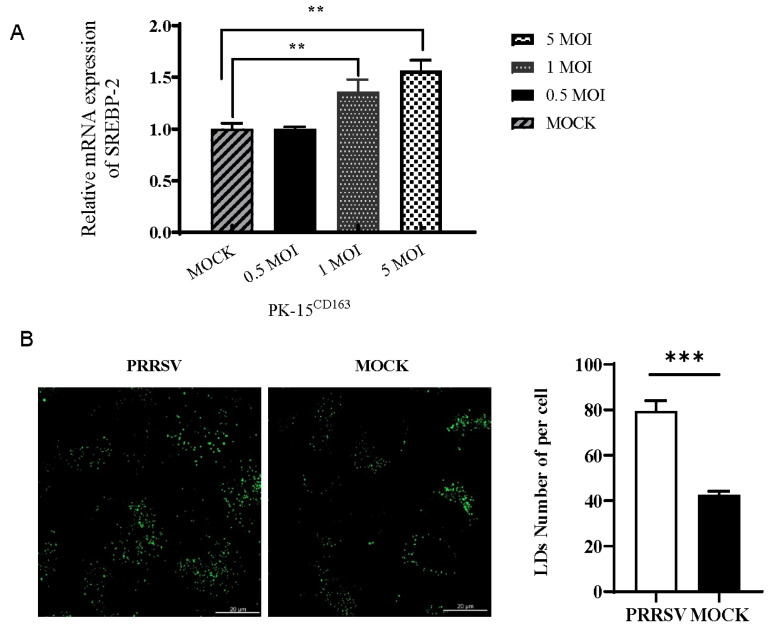
PRRSV infection promotes SREBP2 mRNA expression and lipid droplet increase. (**A**) PRRSV infection upregulates SREBP2 mRNA expression. (**B**) PRRSV infection promotes lipid droplet (Green) increase (Figure S1). (** *p* < 0.01, *** *p* < 0.001).

Changes in lipid metabolism are a common feature of various viral infections, among which the SREBP family serves as the core switch for maintaining cellular lipid homeostasis [33,34,35]. Based on quantitative RT-PCR analysis, we found that PRRSV infection at multiplicities of infection of 1.0 and 5.0 significantly upregulated *SREBP2* mRNA expression in PK15^CD163^ cells (*p* < 0.01), indicating that the expression of *SREBP2* was correlated with the number of PRRSVs (Figure 3A). Meanwhile, the number of lipid droplets in cells was significantly increased (*p* < 0.01) after cells were infected with PRRSV (Figure 3B).

### 3.2. Lipid Metabolite Analysis of Let-7f-5p Regulation of PRRSV Infection

#### 3.2.1. Let-7f-5p Inhibits Lipid Droplet Deposition and PRRSV Replication

**Figure 4:**
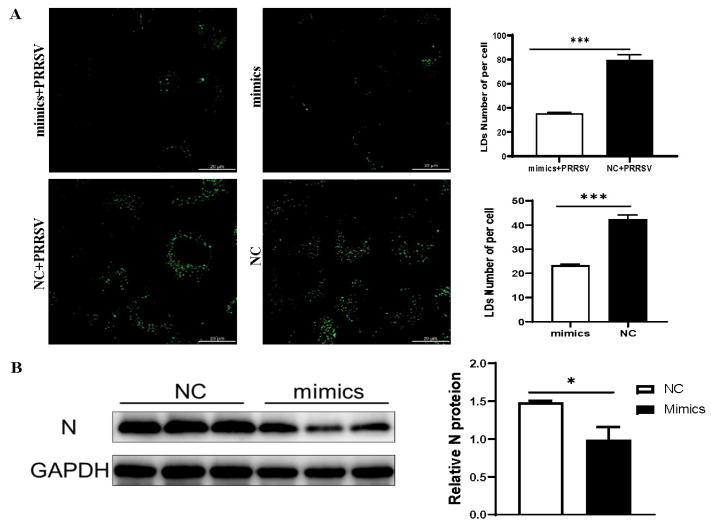
Let-7f-5p suppresses lipid droplet formation and PRRSV replication. (**A**) Let-7f-5p inhibits lipid droplet (Green) accumulation (Figure S2). (**B**) Let-7f-5p suppresses viral replication (Figure S3). Note: *** *p* < 0.001; * *p* < 0.05.

A key focus of our subsequent research is to determine whether viral replication can be suppressed by inhibiting host cellular lipid metabolism through the application of exogenous agents. Based on our previous finding that let-7f-5p targets the 3′ UTR of SREBP2 to inhibit its mRNA expression [32], we further investigated the role of let-7f-5p in lipid metabolism and PRRSV replication. Transfection of MARC-145 cells with let-7f-5p mimics significantly reduced lipid droplet accumulation (*p* < 0.001), and a similar trend was observed in PRRSV-infected cells (Figure 4A). Furthermore, Western blot analysis demonstrated that the let-7f-5p mimic also markedly suppressed the expression of the PRRSV N protein (Figure 4B), indicating its potent inhibitory effect on viral replication.

#### 3.2.2. Lipidomics Analysis of Let-7f-5p Regulation in PRRSV-Infected Cells

**Figure 5:**
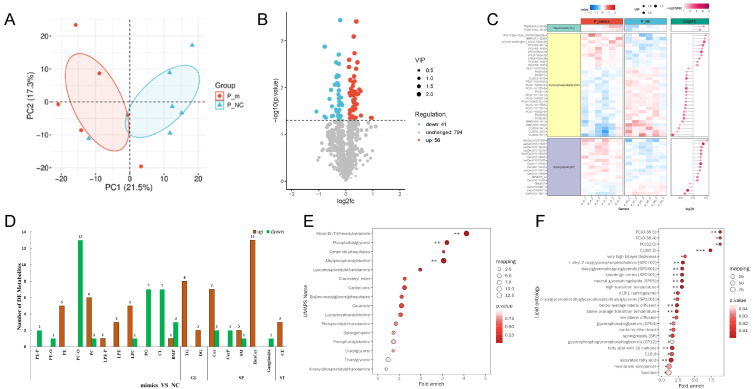
Let-7f-5p regulates lipid-related metabolites. (**A**) Principal component analysis (PCA) plot; (**B**) volcano plot. (**C**) Heatmap. (**D**) Statistical classification of Let-7f-5p-regulated lipid-related compounds. (**E**) Functional classification enrichment based on LIPID MAPS. (**F**) Lipid enrichment analysis using LION. (* *p* < 0.05, ** *p* < 0.01, *** *p* < 0.001).

Lipidomics analysis revealed significant differences in metabolite expression between let-7f-5p mimics-treated and control groups in PRRSV-infected PK15^CD163^ cells (Figure 5A). Using the criteria of *p* < 0.05, VIP > 1, and|log2FC| > 0, we identified 97 differentially expressed lipid compounds, comprising 56 upregulated and 41 downregulated species (Figure 5B). These differential lipid metabolites were primarily classified into two major categories: glycerophospholipids and sphingolipids (Figure 5C). Notably, the abundance of hexosylceramides (HexCers), TG, and Cer showed significant upregulation, while PC-O, cardiolipins (CLs), and phosphatidylglycerols (PGs) were markedly downregulated (Figure 5D). Functional enrichment analysis using the LIPID MAPS database demonstrated that let-7f-5p overexpression-induced lipid metabolites were predominantly enriched in mono-/di-/trihexosylceramides, phosphatidylglycerols, alkylphosphatidylcholines, lysophosphatidylethanolamines, cholesteryl esters, and CL (Figure 5E). LION enrichment analysis further revealed that these differential metabolites were mainly associated with cellular signaling pathways involving phosphatidylcholines and sphingolipids; membrane composition, including 1-alkyl,2-acylglycerophosphocholines and iacylglycerophosphoglycerols; membrane stability and fluidity parameters, showing below-average lateral diffusion and above-average transition temperatures, and metabolic pathways of saturated C16 fatty acids (Figure 5F).

### 3.3. Let-7f-5p Inhibits Viral Replication by Interfering with PRRSV-Induced Lipid Metabolic Reprogramming

**Figure 6:**
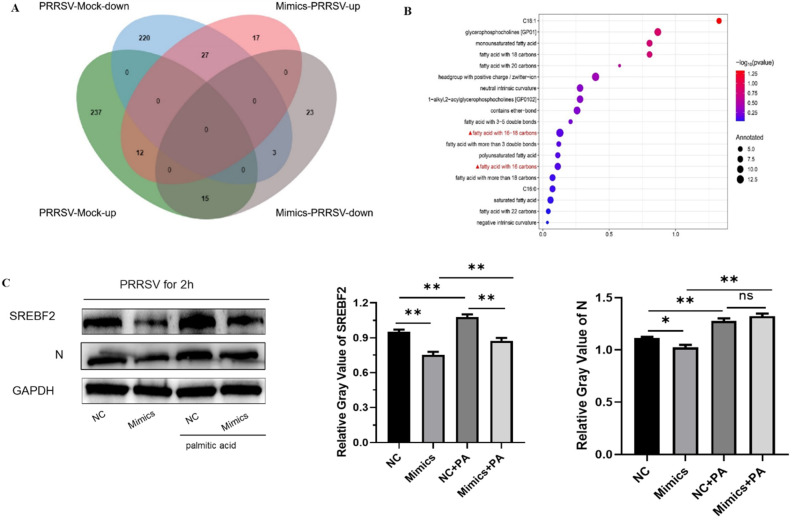
Wenn diagram of differentially expressed lipid metabolites and enrichment pathways. (**A**) Wenn diagram analysis. (**B**) Lipid metabolite enrichment pathway. (**C**) Palmitic acid (PA) restores *SREBP2* and PRRSV N protein expression (Figure S4). (* *p* < 0.05; ** *p* < 0.01).

Comparative analysis of differential lipid metabolites between PRRSV-infected and uninfected cells, as well as PRRSV-infected cells overexpressing let-7f-5p, revealed 15 lipid metabolites that were significantly upregulated upon PRRSV infection but downregulated following let-7f-5p overexpression in infected cells (Figure 6A), specifically, LPC(20:2)[sn2], BMP(18:1-18:1), PC(O-16:0/18:1), PC(O-16:0/20:1), CL(32:2-34:3), PC(O-16:0/20:3), PC(O-16:0/22:1), PC(O-16:0/22:4), PC(O-18:1/16:0), PC(O-16:0/22:5)_n3, PC(O-16:0/22:5)_n6, PC(O-18:1/16:1), PC(O-18:1/18:1), PE(O-18:1/18:1), and PE(P-18:0/22:6). These lipids were primarily enriched in metabolic pathways involving phosphatidylcholines, monounsaturated fatty acids, C18 fatty acids, and C16 fatty acids (Figure 6B). Rescue experiments demonstrated that supplementation with 20 μM palmitic acid (C16:0) significantly restored SREBP2 protein expression and concurrently upregulated PRRSV N protein expression (Figure 6C). These results confirm that let-7f-5p suppresses viral proliferation by downregulating SREBP2 to modulate lipid metabolism.

### 3.4. Effects of Let-7f-5p on the Expression Levels of Inflammatory Factors TNF-α and IL-10

**Figure 7:**
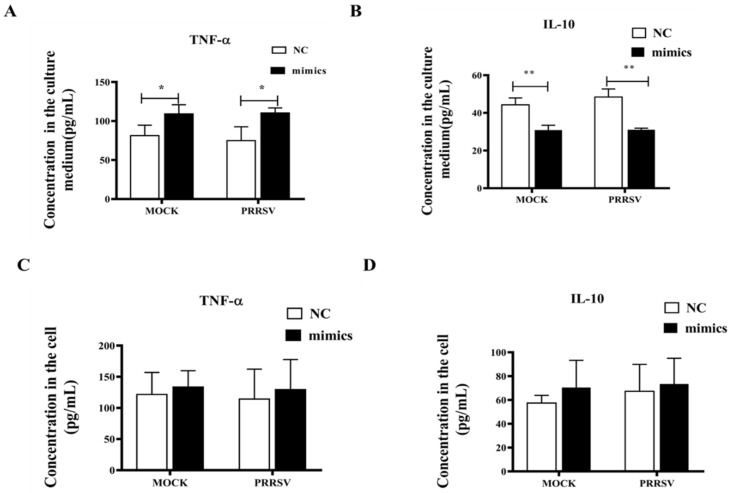
Analysis of the effect of let-7f-5p on inflammatory factors. (**A**) TNF-α contents in cell culture medium. (**B**) IL-10 contents in cell culture medium. (**C**) The contents of TNF-α in cells; (**D**): The contents of IL-10 in cells. (* *p* < 0.05; ** *p* < 0.01).

ELISA results demonstrated that overexpression of let-7f-5p mimics significantly increased TNF-α levels (*p* < 0.05) in the culture medium of both mock-treated (MOCK) and PRRSV-infected PK15^CD163^ cells (Figure 7A) while significantly decreasing IL-10 levels (*p* < 0.01) (Figure 7B). However, no significant differences were observed in the intracellular expression levels of these two inflammatory factors (Figure 7C,D).

## 4. Discussion

### 4.1. PRRSV Infection Induces Cellular Lipid Metabolic Dysregulation

Porcine alveolar macrophages (PAMs) are the primary target cells for PRRSV, but their inability to proliferate long-term in vitro has limited their application in pathogenetic studies. The PK15 cell lines stably expressing porcine CD163 protein (PK15^CD163^) can be infected by PRRSV strains of different genotypes and are often used for experimental studies of PRRSV [36]. In this study, we found a total of 735 genes that exhibited significant changes in expression based on transcriptomic data from PK15^CD163^ cells infected with PRRSV, with 683 genes upregulated and 52 genes downregulated. The differentially expressed genes were most predominantly enriched in metabolic pathways, which further demonstrated that PRRSV, like other viruses, needs to promote its own replication and survival by regulating host cell metabolism [20,37]. Among these alterations, we found that differentially expressed genes (DEGs) were significantly enriched in lipid metabolism. Lipid metabolism plays a crucial role in viral replication [33]. To rapidly and extensively replicate, viruses exploit cellular lipid signaling and metabolic pathways to create favorable conditions [38,39]. It is worth noting that the significant upregulation of SREBP2 expression is dose-dependent with the diversity of viral infections (*p* < 0.01). In addition, we found that lipid metabolism was significantly altered in PK15^CD163^ cells after infection with PRRSV by broad-targeted lipidomic analysis, especially PE-*p* and PE-O, which were significantly increased, reflecting that the viral infection altered cellular metabolism to satisfy the energy requirements for viral replication and the biosynthetic precursors for viral replication. Yang et al. demonstrated that PRRSV modulates lipid metabolic pathways to promote lipid droplet accumulation [40]. TGs are the main form of intracellular energy storage [41]. Both HCV and dengue virus have been reported to promote the assembly and release of viral particles by increasing TG synthesis [42]. PE is an important component of cell membranes and is involved in the regulation of membrane structure, stability, and fluidity [43]. In addition, PE protects the viral infection during the cell membranes from attack by the host immune system [44].

In contrast, the abundance of PC, Cer, LPC and CAR was significantly downregulated after virus infection. PC is the most abundant phospholipid component of cell membranes and is involved in membrane integrity and signaling [45], and its downregulation may lead to alterations in the structure of cell membranes, which in turn may affect membrane-associated signaling and viral entry processes. In addition, PC is a precursor of lipid signaling molecules, and its downregulation may affect the activity of downstream signaling pathways. Cer, as a key intermediate of sphingolipid metabolism, is involved in various biological processes such as apoptosis, autophagy, and inflammatory response [46], and its downregulation may be involved in inhibiting apoptotic pathways, which prolongs the survival time of the infected cells and provides a longer window period for viral replication. Also, Cer is involved in regulating microdomain structures of cell membranes [46], and its downregulation may alter the microenvironment for viral entry and assembly.

LPC is a hydrolysis product of PC; it has proinflammatory and immunomodulatory effects, and its downregulation may inhibit the inflammatory response of host cells, thereby helping viruses evade recognition and clearance by the immune system [47]. In addition, LPC is involved in regulating cell membrane fluidity, and its downregulation may also further alter the physical properties of cell membranes, affecting viral entry and release processes. CARs, as an intermediate metabolite in fatty acid metabolism, participate in the mitochondrial β-oxidation process [48]. Their downregulation likely reflects suppressed fatty acid metabolism following PRRSV infection, resulting in reduced cellular energy supply. This lipid metabolic dysregulation may provide additional lipid resources for viral replication while simultaneously compromising host cell antiviral responses by altering cellular energy homeostasis [48]. In conclusion, PRRSV infection induces significant dysregulation of cellular lipid metabolism through multiple mechanisms, including alterations in membrane structure, signal transduction, and energy metabolism. These changes are further supported by LION enrichment analysis, which revealed substantial modifications in both lipid species composition and their physicochemical properties following viral infection.

### 4.2. Let-7f-5p Inhibits Viral Replication by Regulating Cellular Lipid Metabolism

Let-7f-5p, encoded by clusters 2 and 4 on chromosomes 9 and X, has been implicated in various physiological processes such as embryonic development, skeletogenesis, vascular function, and neuroregulation, as well as exhibiting antitumor and anti-inflammatory properties [49,50]. Our group previously confirmed that let-7f-5p directly targets SREBP2 and significantly suppresses the expression of SREBP2 and other lipogenic genes [32]. Lipidomic profiling revealed that let-7f-5p altered the production of 97 differential lipid compounds, predominantly glycerophospholipids and sphingolipids, such as PC, CL and PG, all of which are important components of cellular and organelle membranes. PC is uniquely required for lipoprotein assembly and secretion [45,51], and CL is a mitochondria-specific class of phospholipids that plays a key role in maintaining the curvature of mitochondrial inner membrane cristae [52,53]. These findings suggest that let-7f-5p not only regulates cellular energy metabolism but also modifies membrane architecture.

Comparison analysis of differential lipid metabolites before and after PRRSV infection and before and after let-7f-5p transfection showed that 15 differential lipid metabolites were upregulated after PRRSV infection but downregulated in expression after the addition of let-7f-5p mimics, which were mainly enriched in the metabolic pathways such as phosphatidylcholine, monounsaturated fatty acids, fatty acids of 16–18 carbon atoms, 16 carbon atom fatty acids, and other metabolic pathways, which are hypothesized to be the main lipid metabolites involved in viral replication. And it was also confirmed by the addition of palmitic acid (fatty acid of 16 carbon atoms), which upregulated the inhibitory regulatory effect of let-7f-5p on SREBP2 and PRRSV replication. This discovery reveals the important role of lipid metabolites in the proliferation of PRRSV. Research has found that lysophosphatidic acid binds to its receptor LPAR1 and then induces the activation of Rho-associated coiled-coil containing protein kinase 1/2 (ROCK1/2), thereby inhibiting the interferon (IFN)-mediated host response against viruses such as Zika virus and SARS-CoV-2 [54]. Zhang et al. found that inhibiting LPA exhibits an antiviral effect during PRRSV infection [55]. In the future, more lipid metabolites need to be explored as potential targets for PRRSV prevention.

### 4.3. Effect of Let-7f-5p on Gene Expression of Inflammatory Factors TNF-α and IL-10

TNF-α is a cytokine produced by a variety of cells, including activated macrophages, T-lymphocytes, B-lymphocytes, natural killer cells, astrocytes, endothelial cells, smooth muscle cells, some tumor cells, and epithelial cells [56], which promotes the infiltration and activation of inflammatory cells and enhances the antiviral immune response. Previous studies have demonstrated its ability to inhibit the replication of vesicular stomatitis virus, encephalomyocarditis virus, and herpes simplex virus while preventing virus-induced cytopathic effects [57]. TNF-α is the host’s first line of defense against influenza viral infections [58], and in the present study, we found that overexpression of let- 7f-5p was able to significantly upregulate the expression level of TNF-α in the culture medium. It suggests that let-7f-5p inhibits PRRSV replication and may also be related to the up-regulation of the TNF-α signaling pathway. IL-10 is also a potent anti-inflammatory cytokine, and its level was decreased in let-7f-5p-treated medium of PK15^CD163^ cells infected with PRRSV, suggesting that let-7f-5p may have a role in balancing inflammatory and anti-inflammatory factors. These results indicate that let-7f-5p can influence the production of inflammatory factors, which may be involved in the antiviral immune process, but its specific molecular mechanism needs further exploration.

## 5. Conclusions

The results of multi-omics analysis revealed that PRRSV infection induces host lipid metabolic remodeling, characterized by significant upregulation of triglycerides and sphingolipids and downregulation of phosphatidylcholines and cardiolipins. By employing SREBP2-targeting miRNA let-7f-5p and palmitic acid (C16:0) supplementation, we demonstrated that modulation of lipid metabolism can effectively inhibit PRRSV replication. These findings not only enrich the theoretical framework of virus–host interactions at the scientific level but also provide practical insights for developing novel antiviral strategies through exogenous lipid metabolite supplementation and guiding future PRRSV-resistant breeding programs. Future studies could further explore the specific molecular mechanisms by which key lipid metabolites regulate PRRSV proliferation.

## Figures and Tables

**Table 1:** List of primers used in qRT-PCR.

Primers	Primer Sequence (5′-3′)
SUS-GAPDH-F	CGTCCCTGAGACACGATGGT
SUS-GAPDH-R	GCCTTGACTGTGCCGTGGAAC
SREBP2-F	TGTGGAGCAGCCTCAATGTC
SREBP2-R	TTTTGCCAGAGCACTGTCCG

**Table 2:** The information of the antibody.

Antibody	Catalog Number (Manufacture)	Working Dilution
SREBP2	28212-1-AP (proteintech)	WB(1:1000)
PRRSV Nucleocapsid protein	GTX129270 (GeneTex)	WB(1:2000)
GAPDH	10494-1-AP (proteintech)	WB(1:5000)

## Data Availability

The original contributions presented in the study are included in the article, further inquiries can be directed to the corresponding authors.

## Supplementary Materials

The following supporting information can be downloaded at: https://www.mdpi.com/article/10.3390/vetsci12121176/s1, Figure S1: PRRSV infection promotes lipid droplet (Green) increase. Figure S2: Let-7f-5p inhibits lipid droplet (Green) accumulation. Figure S3: Let-7f-5p suppresses viral replication. Figure S4: Palmitic acid (PA) restores SREBP2 and PRRSV N protein expression.
