# Supplementary figures and images for: Let-7f-5p Inhibits PRRSV Replication by Regulating Lipid Metabolic Reprogramming in Infected Cells
Source: Vet Sci. 2025 Dec 10;12(12):1176. doi: 10.3390/vetsci12121176 (PMC12737658; doi:10.3390/vetsci12121176)

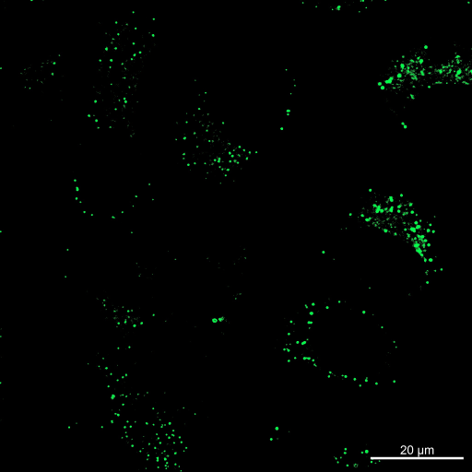

Supplement: Supplementary file 1 [file vetsci-12-01176-s001.zip › Figure S1/MOCK.tif]

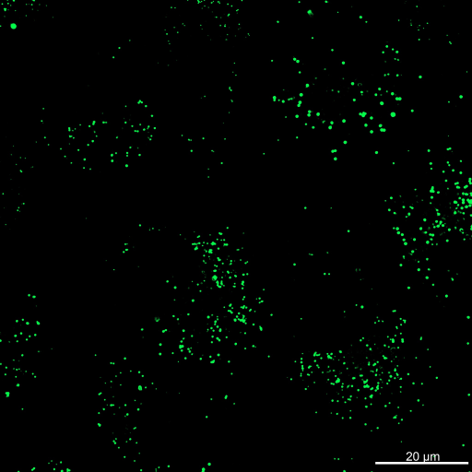

Supplement: Supplementary file 1 [file vetsci-12-01176-s001.zip › Figure S1/PRRSV.tif]

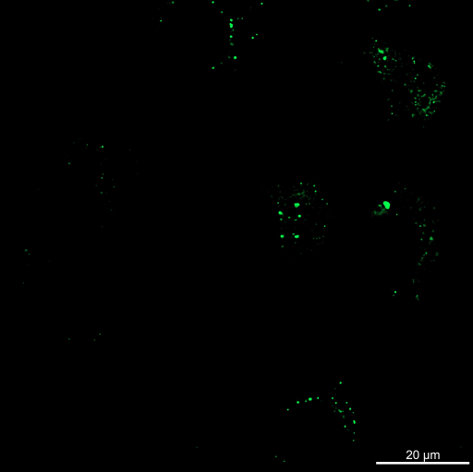

Supplement: Supplementary file 1 [file vetsci-12-01176-s001.zip › Figure S2/MIMICS PPRSV.tif]

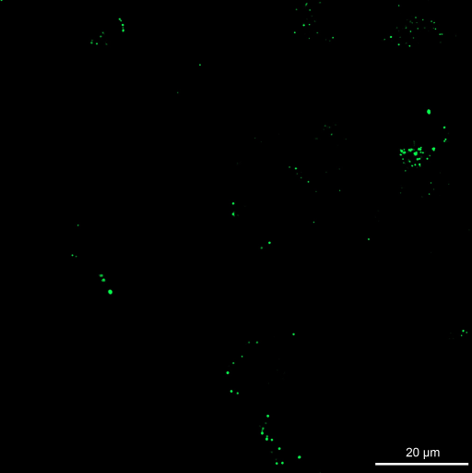

Supplement: Supplementary file 1 [file vetsci-12-01176-s001.zip › Figure S2/MIMICS.tif]

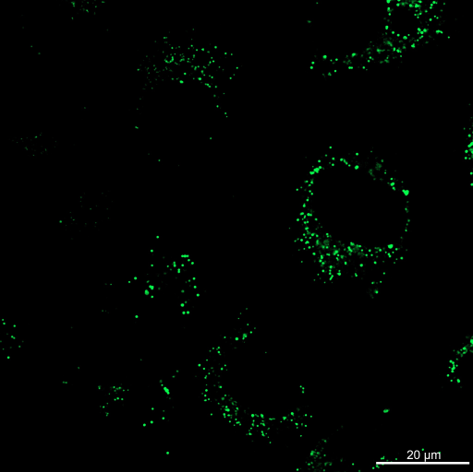

Supplement: Supplementary file 1 [file vetsci-12-01176-s001.zip › Figure S2/NC PPRSV.tif]

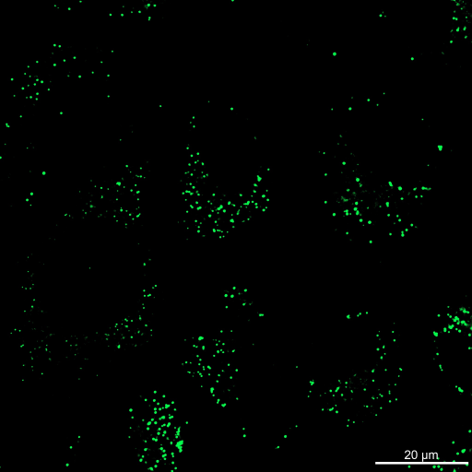

Supplement: Supplementary file 1 [file vetsci-12-01176-s001.zip › Figure S2/NC.tif]

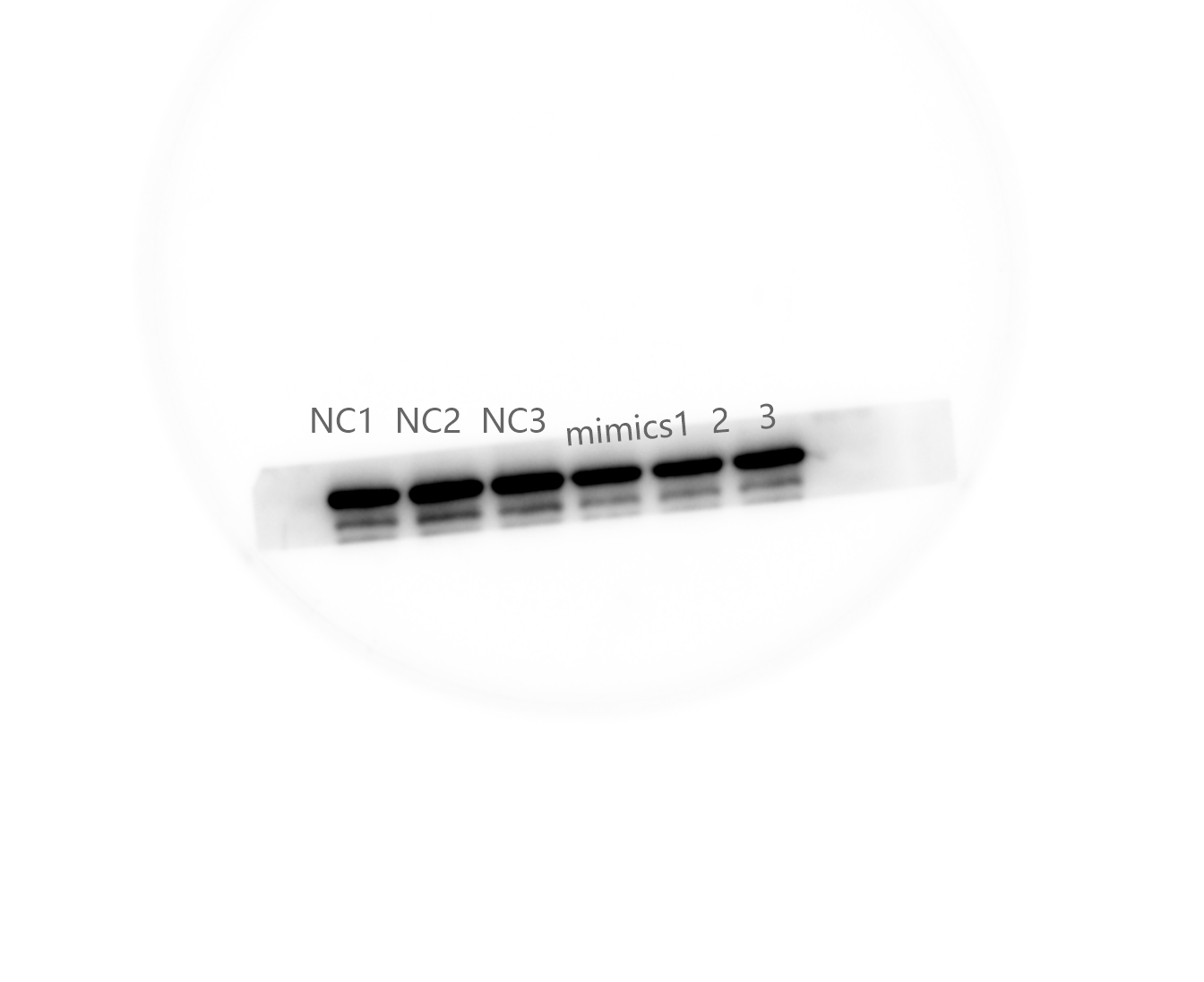

Supplement: Supplementary file 1 [file vetsci-12-01176-s001.zip › Figure S3/GAPDH.Tif]

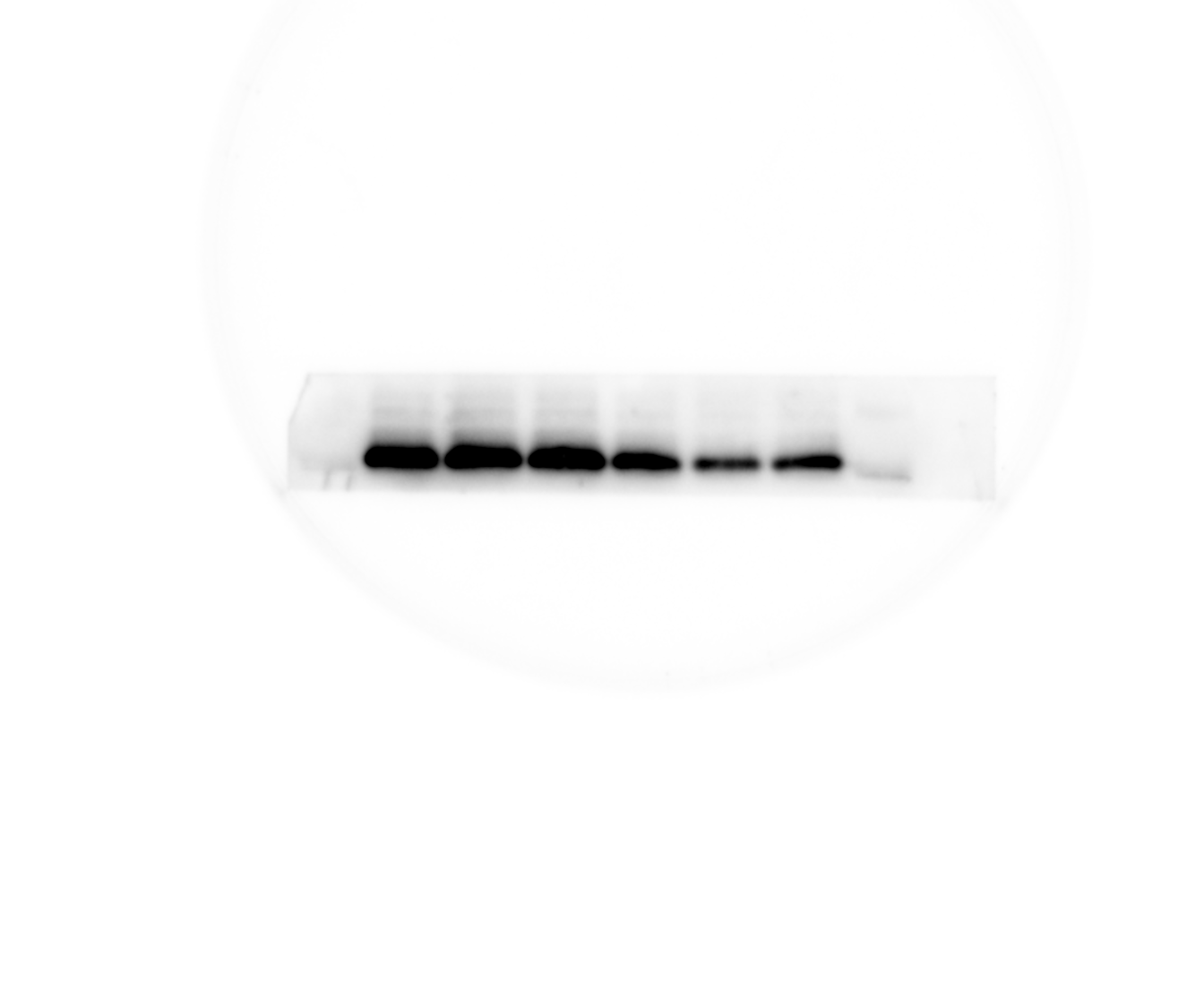

Supplement: Supplementary file 1 [file vetsci-12-01176-s001.zip › Figure S3/N.Tif]

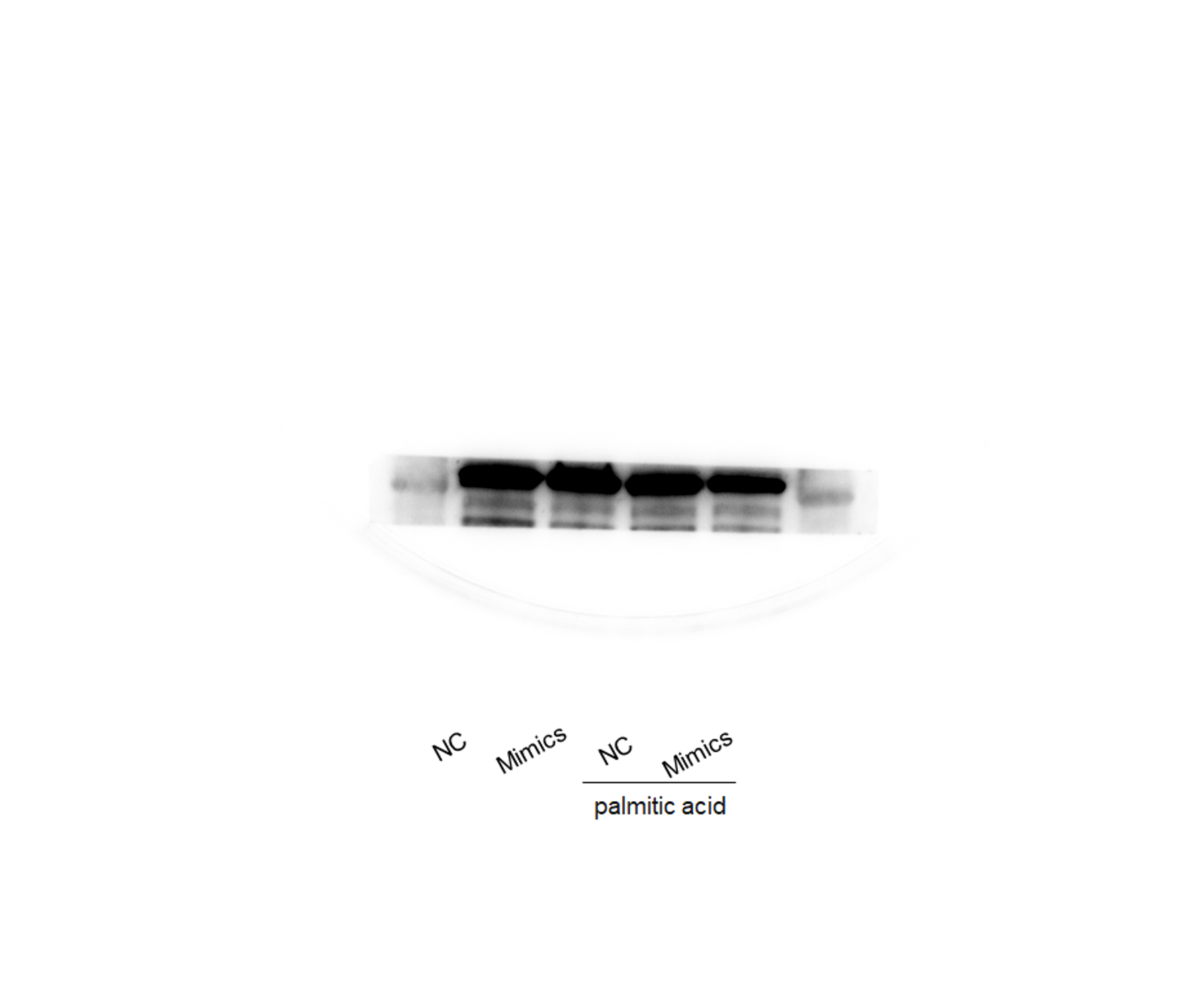

Supplement: Supplementary file 1 [file vetsci-12-01176-s001.zip › Figure S4/GAPDH-1.Tif]

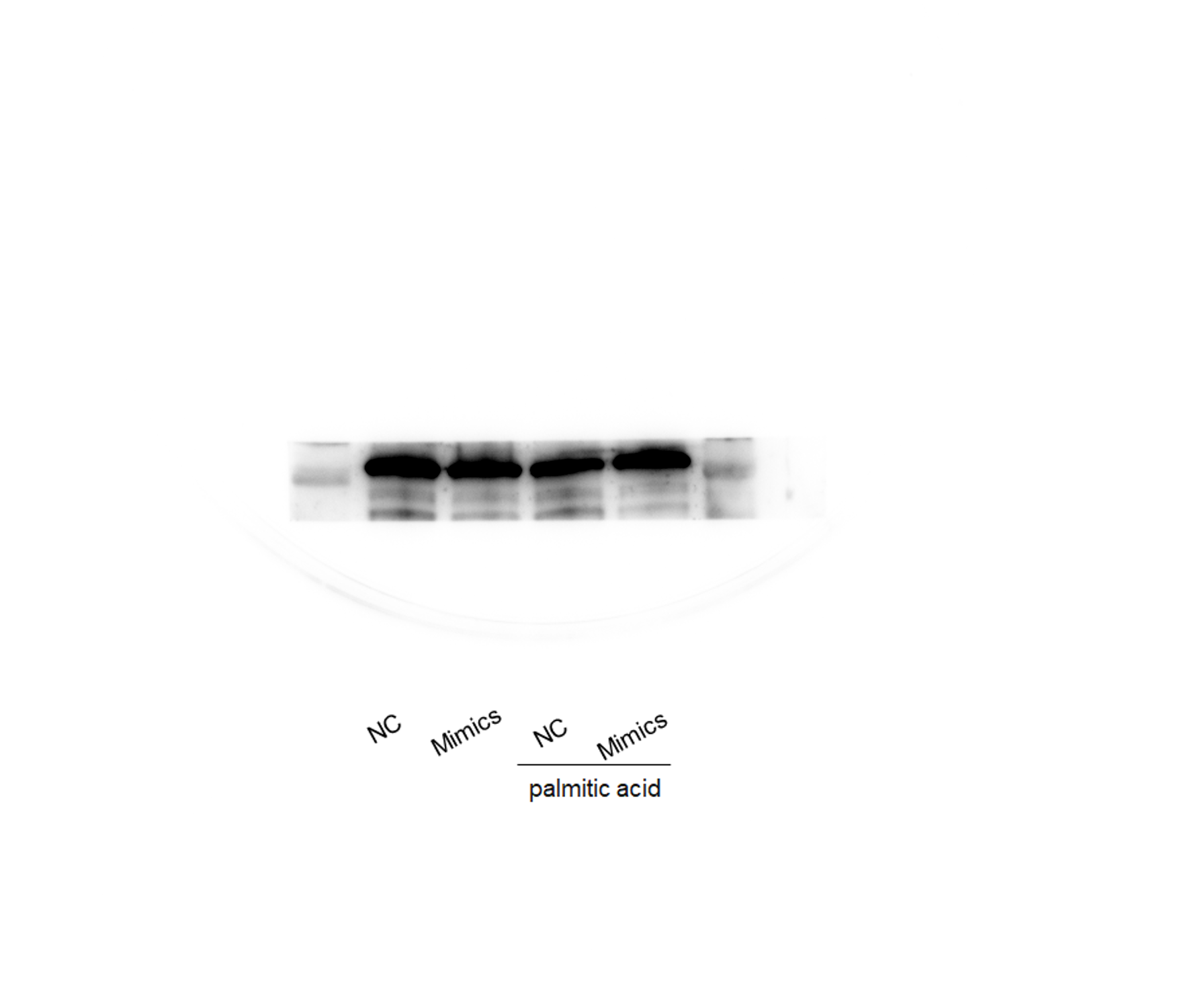

Supplement: Supplementary file 1 [file vetsci-12-01176-s001.zip › Figure S4/GAPDH-2.Tif]

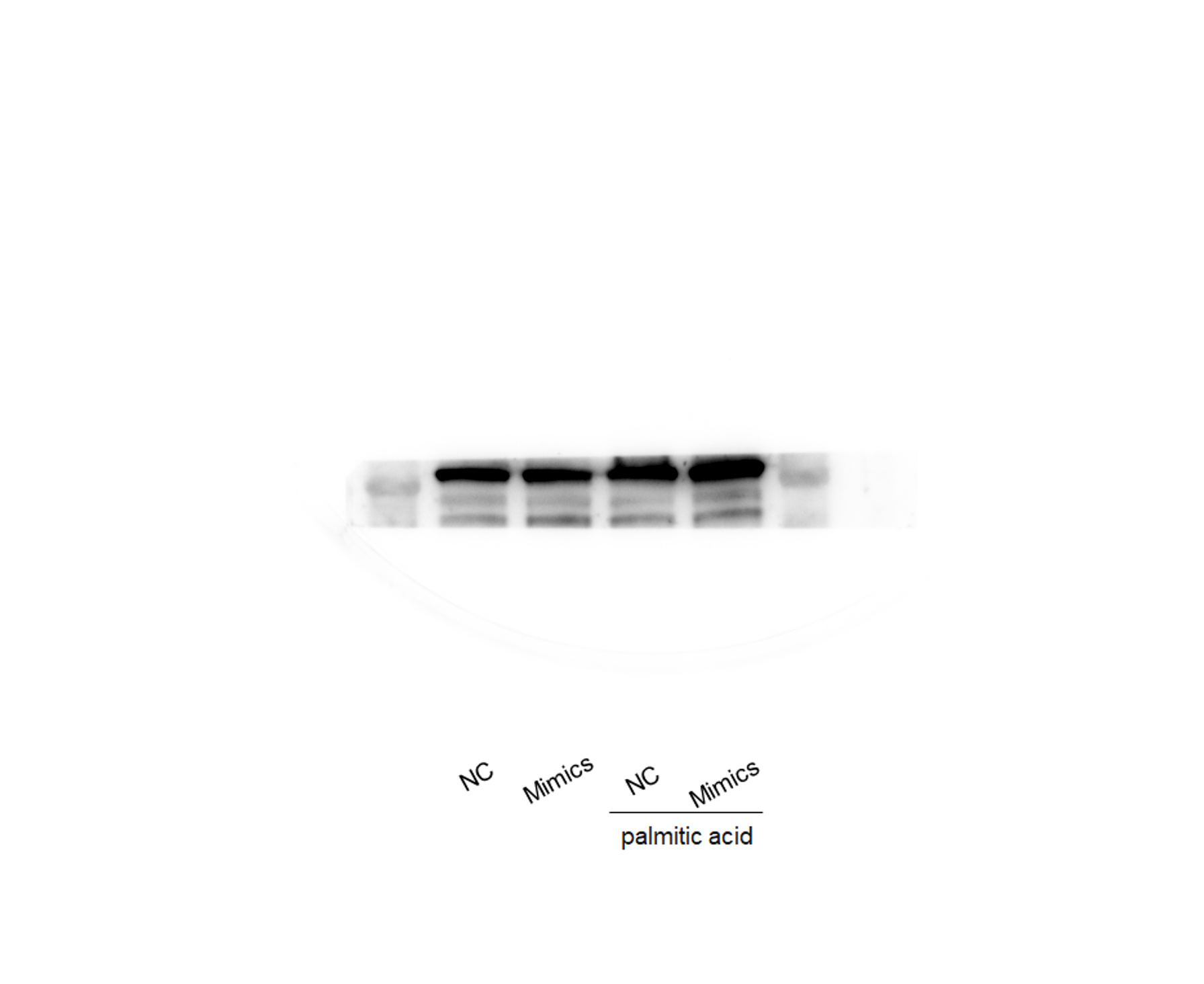

Supplement: Supplementary file 1 [file vetsci-12-01176-s001.zip › Figure S4/GAPDH-3.Tif]

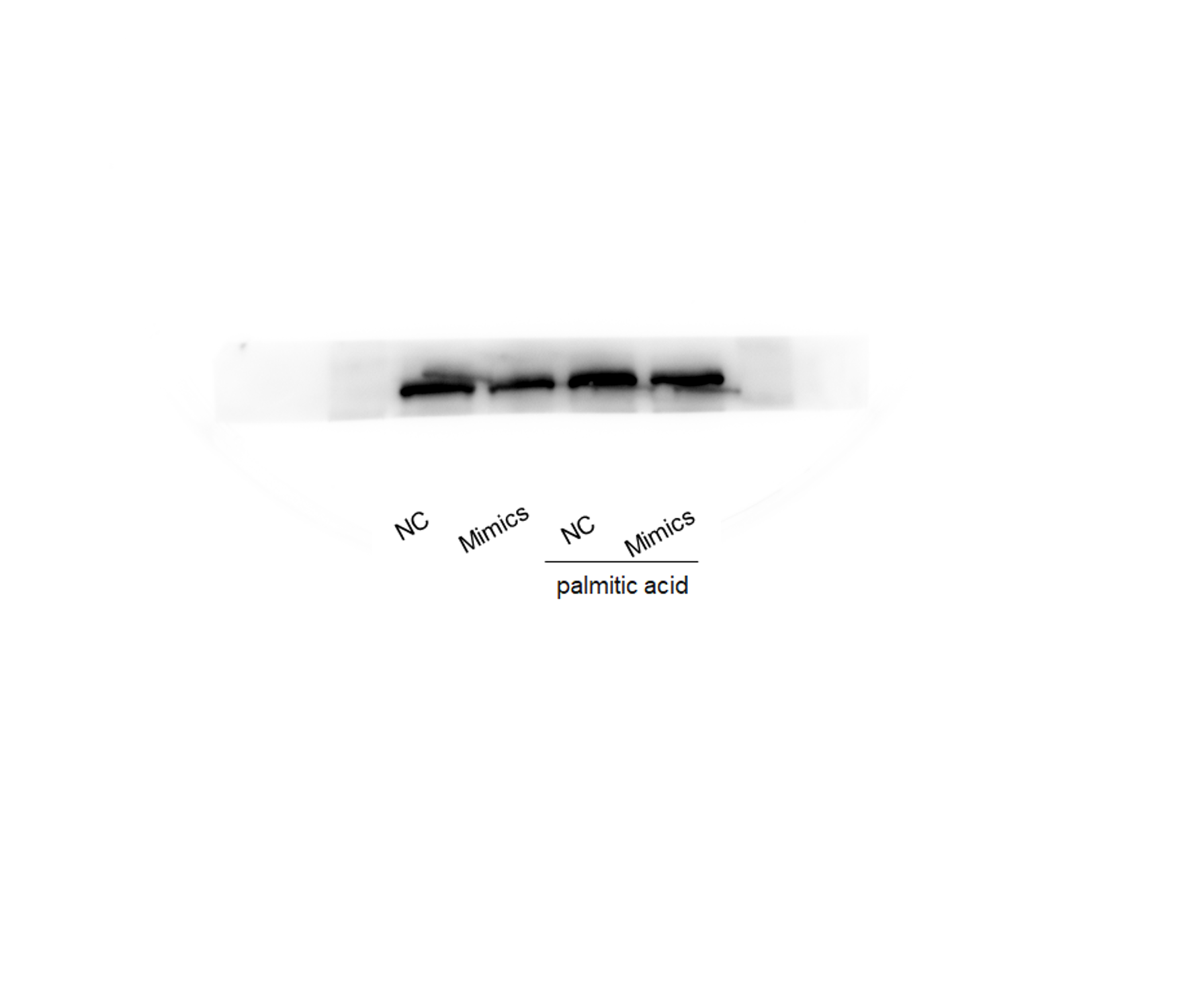

Supplement: Supplementary file 1 [file vetsci-12-01176-s001.zip › Figure S4/N-1.Tif]

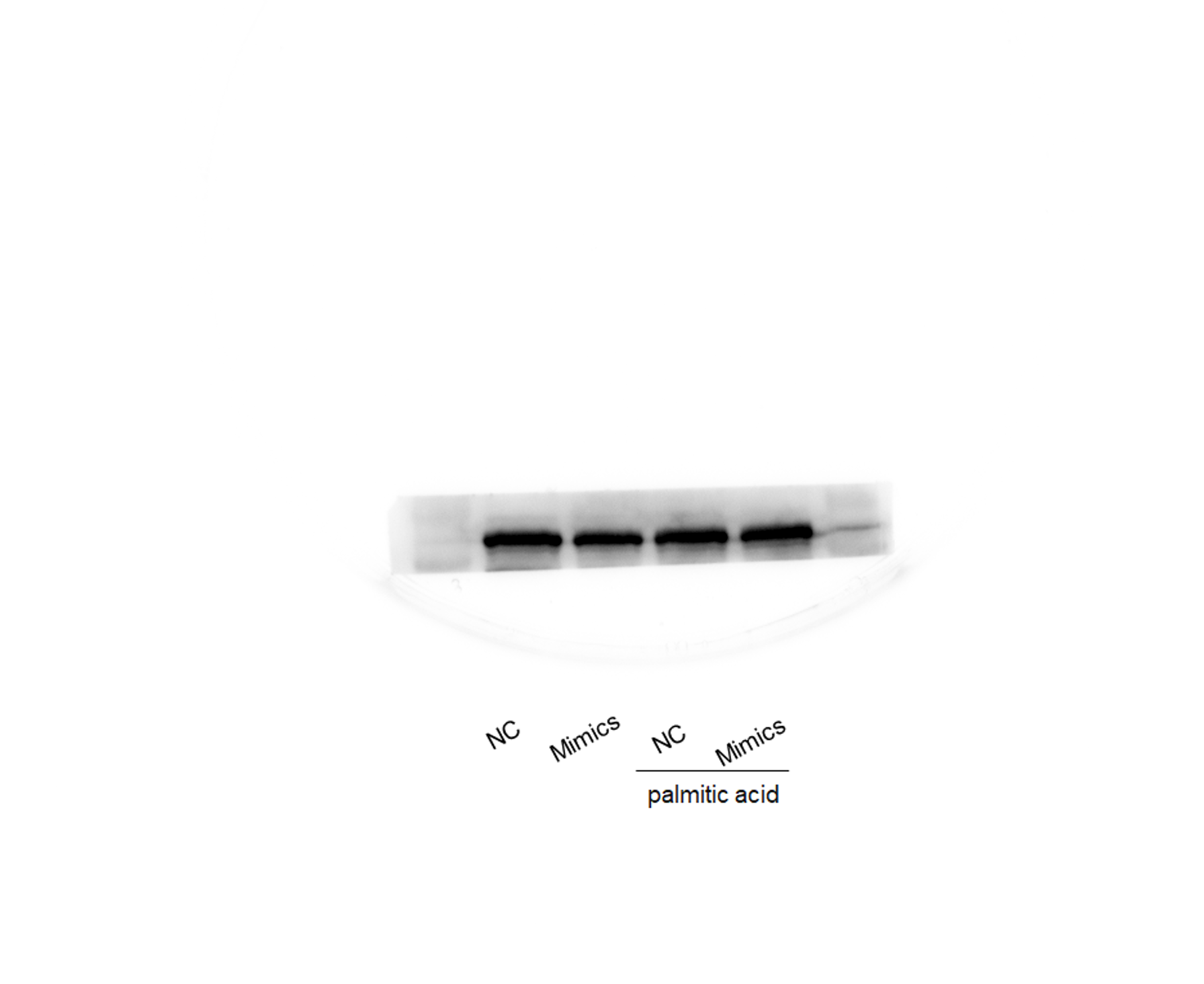

Supplement: Supplementary file 1 [file vetsci-12-01176-s001.zip › Figure S4/N-2.Tif]

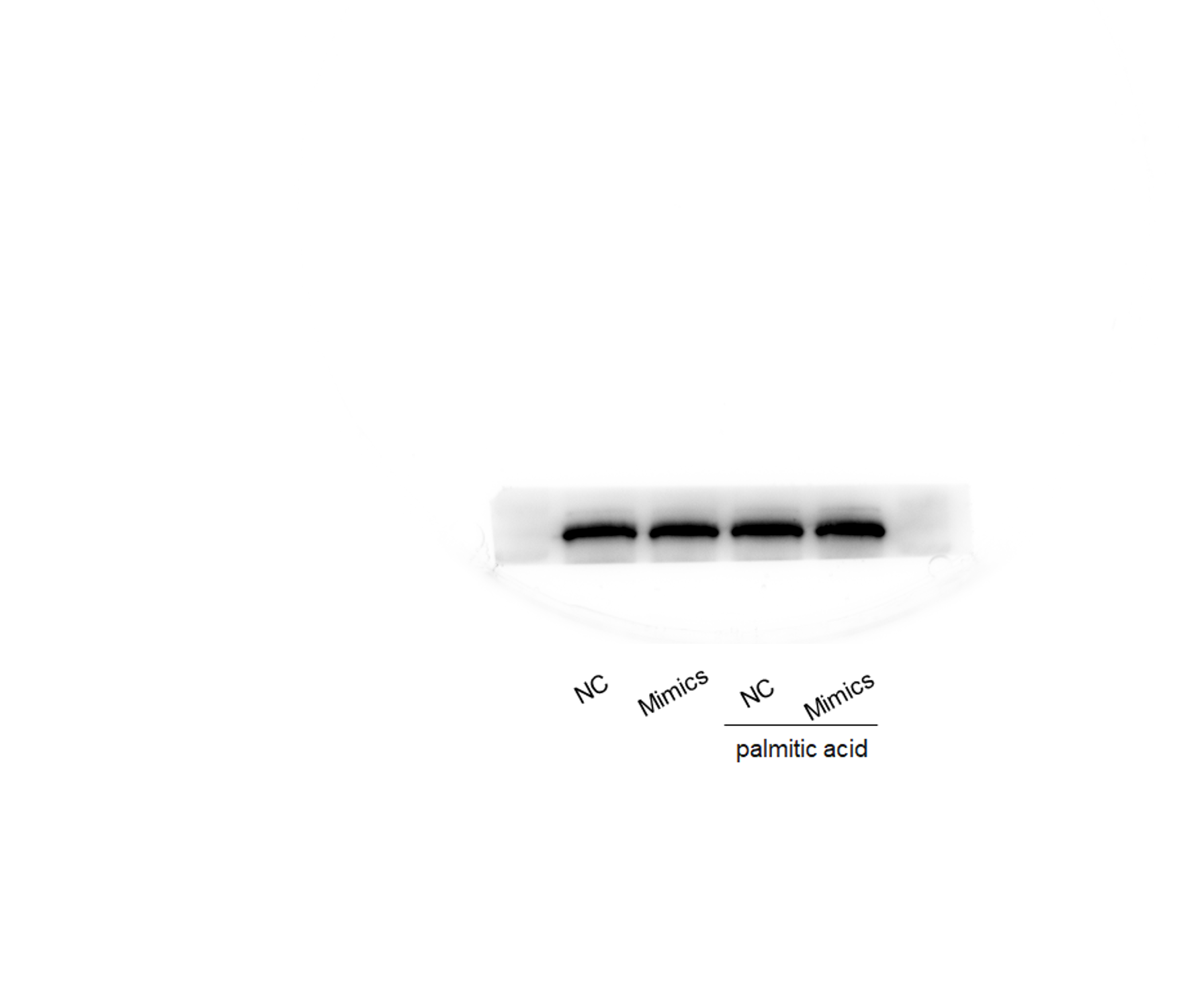

Supplement: Supplementary file 1 [file vetsci-12-01176-s001.zip › Figure S4/N-3-1.Tif]

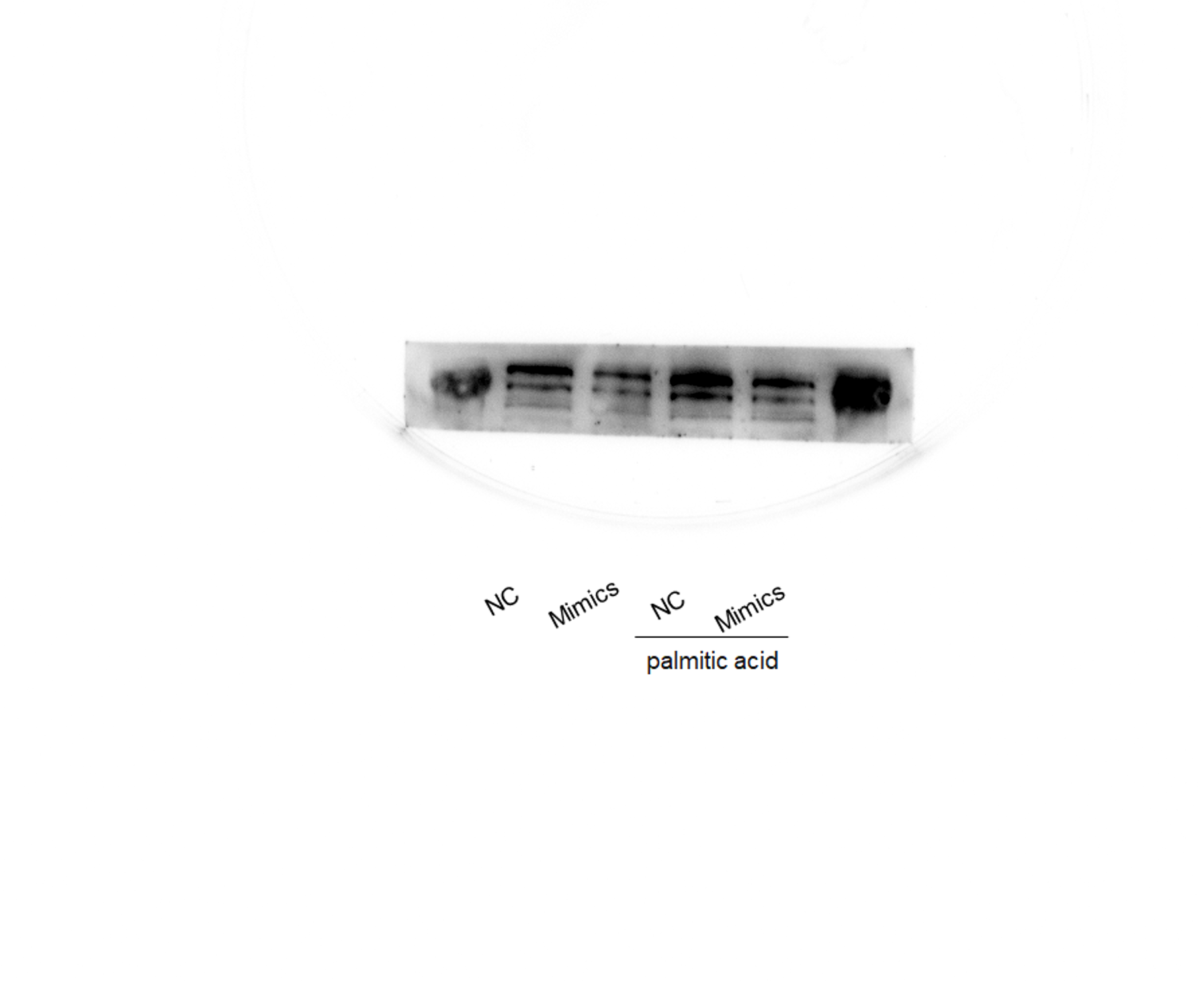

Supplement: Supplementary file 1 [file vetsci-12-01176-s001.zip › Figure S4/SREBF2-1.Tif]

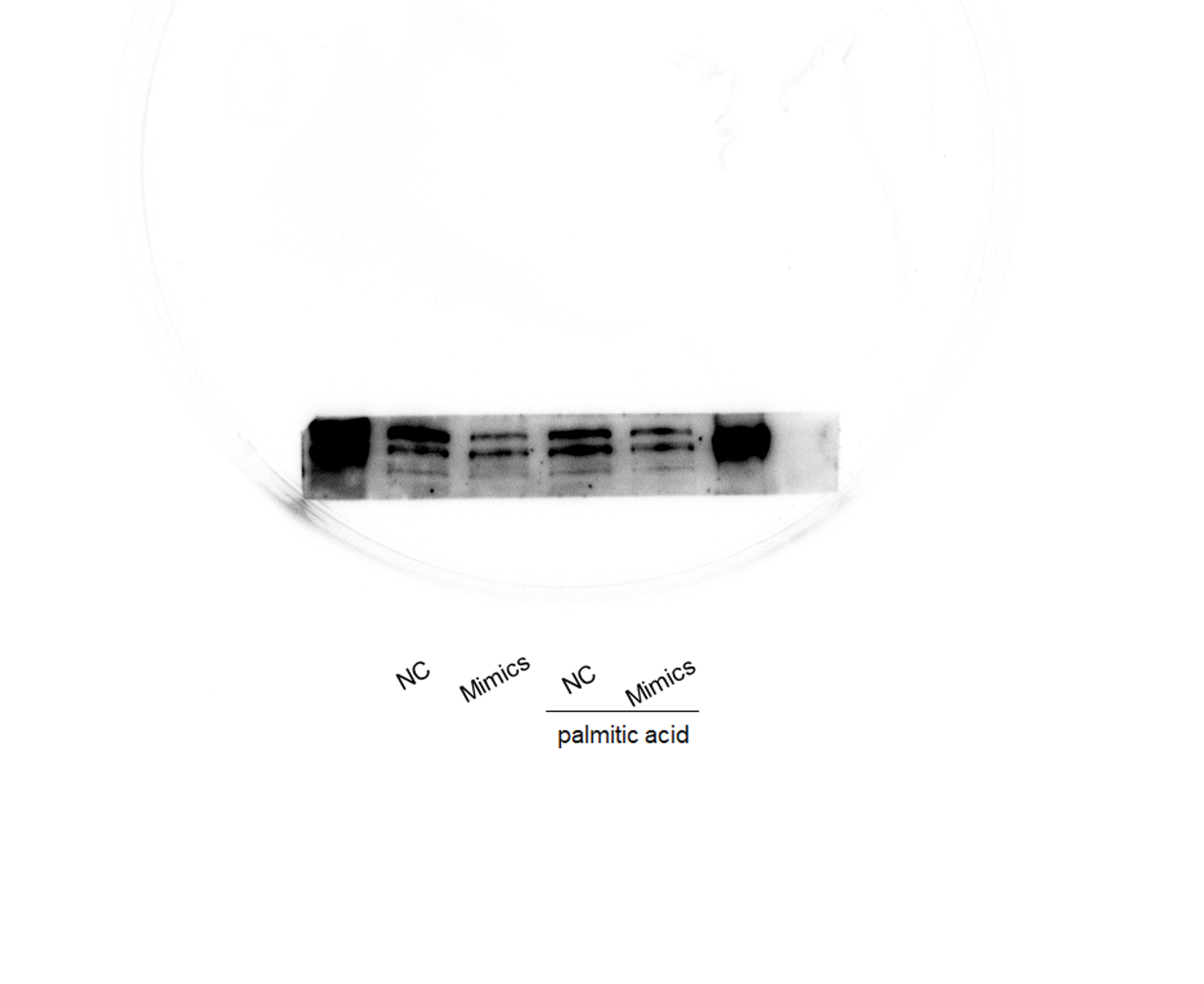

Supplement: Supplementary file 1 [file vetsci-12-01176-s001.zip › Figure S4/SREBF2-2.Tif]

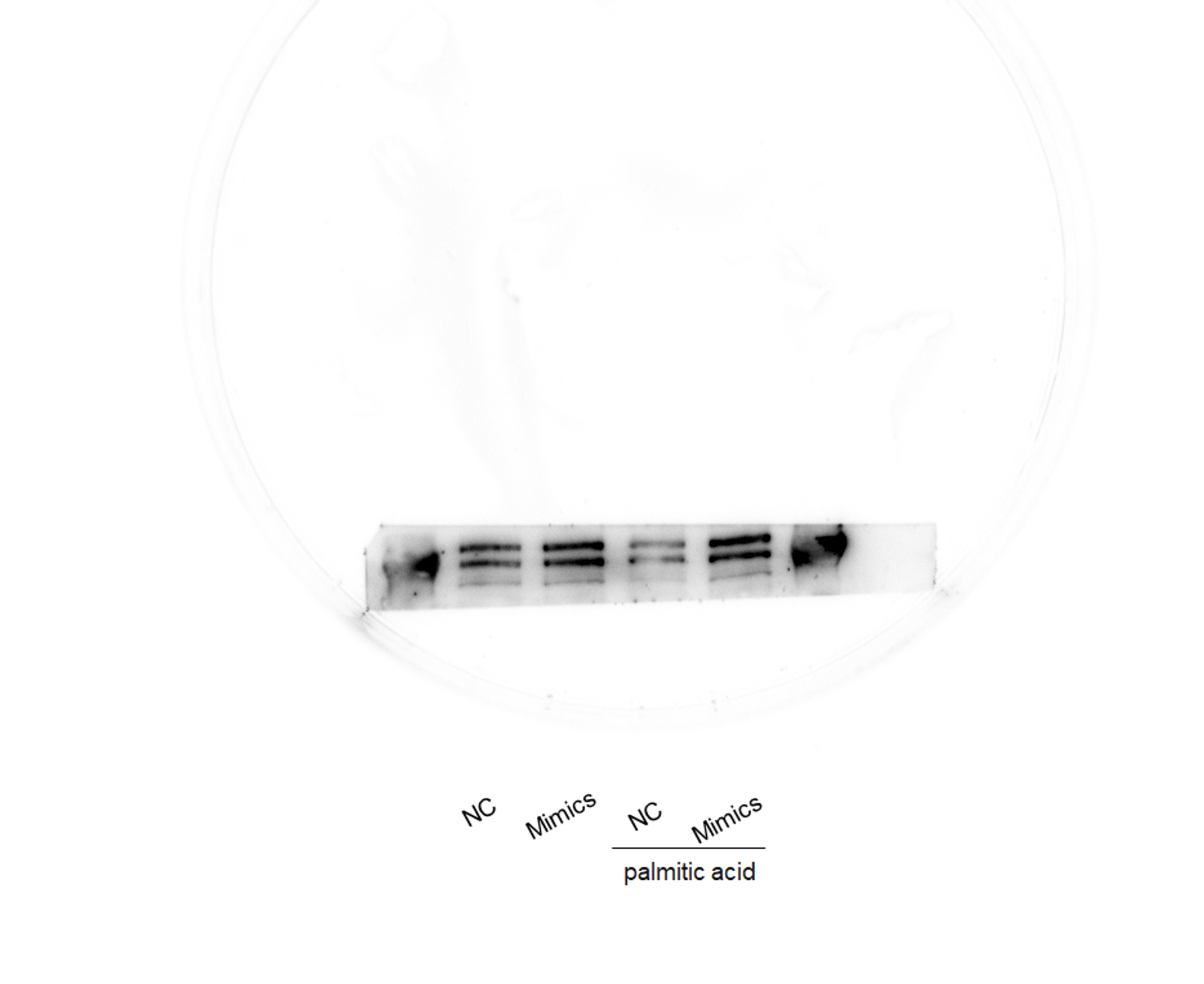

Supplement: Supplementary file 1 [file vetsci-12-01176-s001.zip › Figure S4/SREBF2-3.Tif]
